# Supplementary figures and images for: Intestinal-epithelial LSD1 controls goblet cell maturation and effector responses required for gut immunity to bacterial and helminth infection
Source: PLoS Pathog. 2021 Mar 31;17(3):e1009476. doi: 10.1371/journal.ppat.1009476 (PMC8041206; doi:10.1371/journal.ppat.1009476)

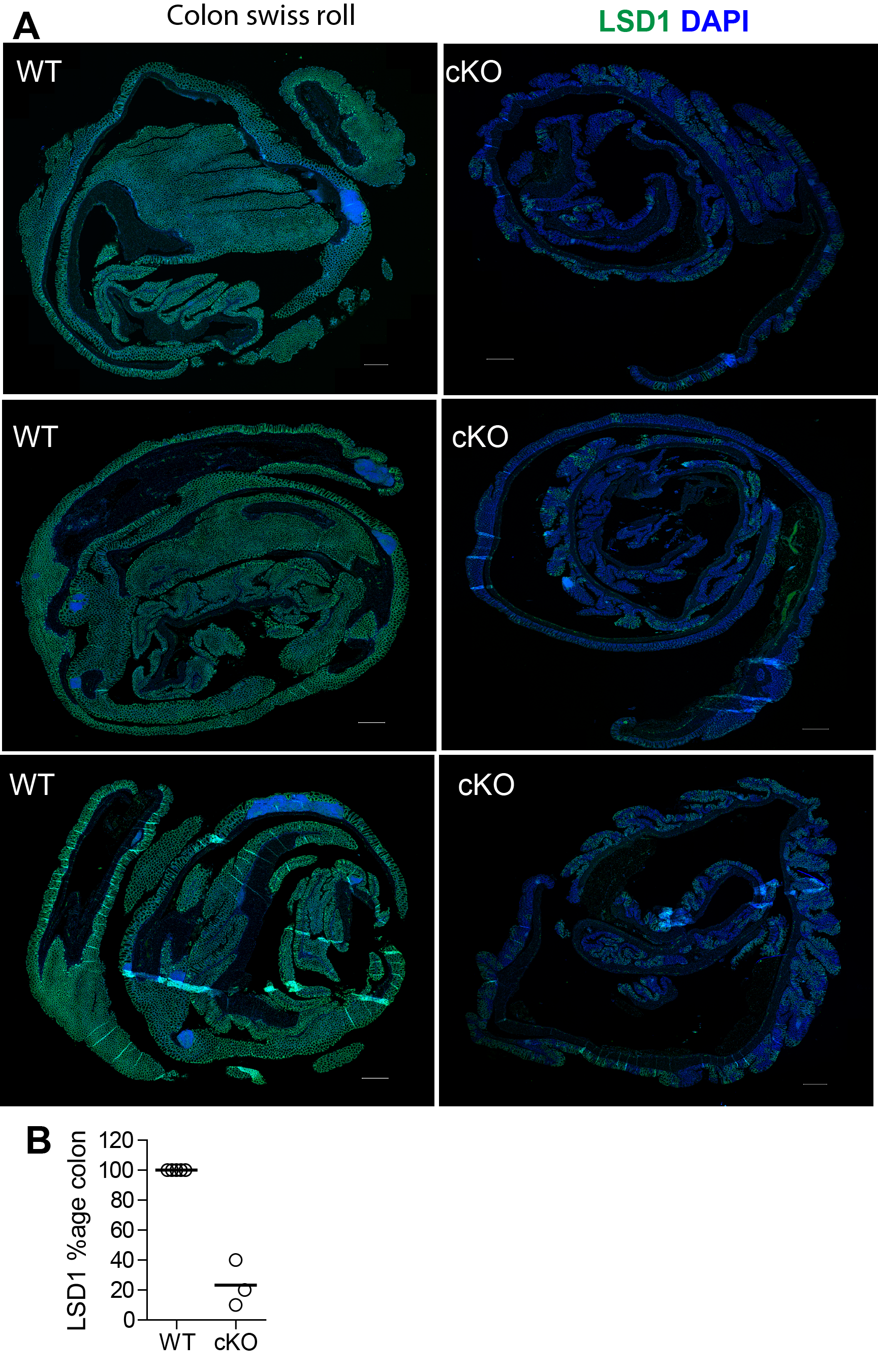

Supplement: S1 Fig — (A) Confocal microscope images of whole colon swiss roles stained for LSD1 (green) expression comparing naive WT and cKO mice. DAPI (blue) was used as a nuclear counterstain. (B) LSD1 expression in WT and cKO colon WT(n = 5) and cKO (n = 3). (TIF) [file ppat.1009476.s001.tif]

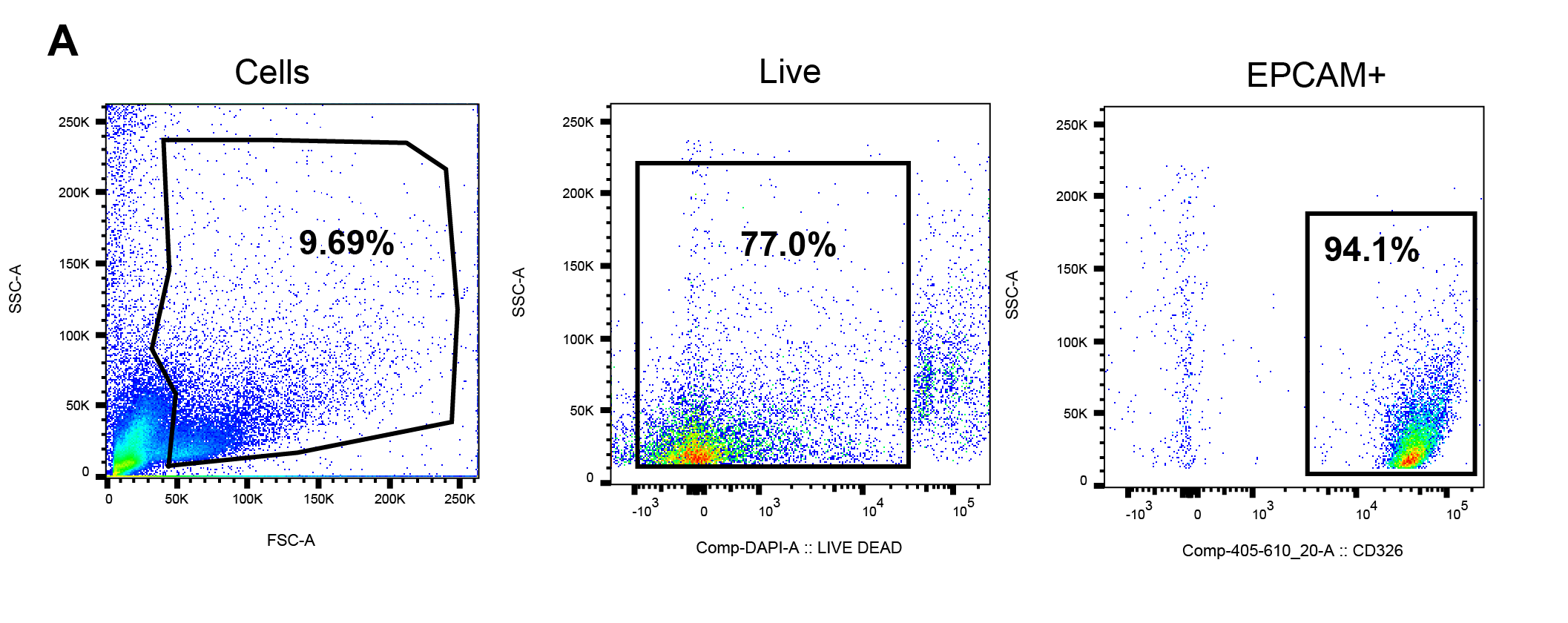

Supplement: S2 Fig — (A) Gating strategy used for flow cytometry plots to determine DAPI- (live) EPCAM+ (epithelial) cells. (TIF) [file ppat.1009476.s002.tif]

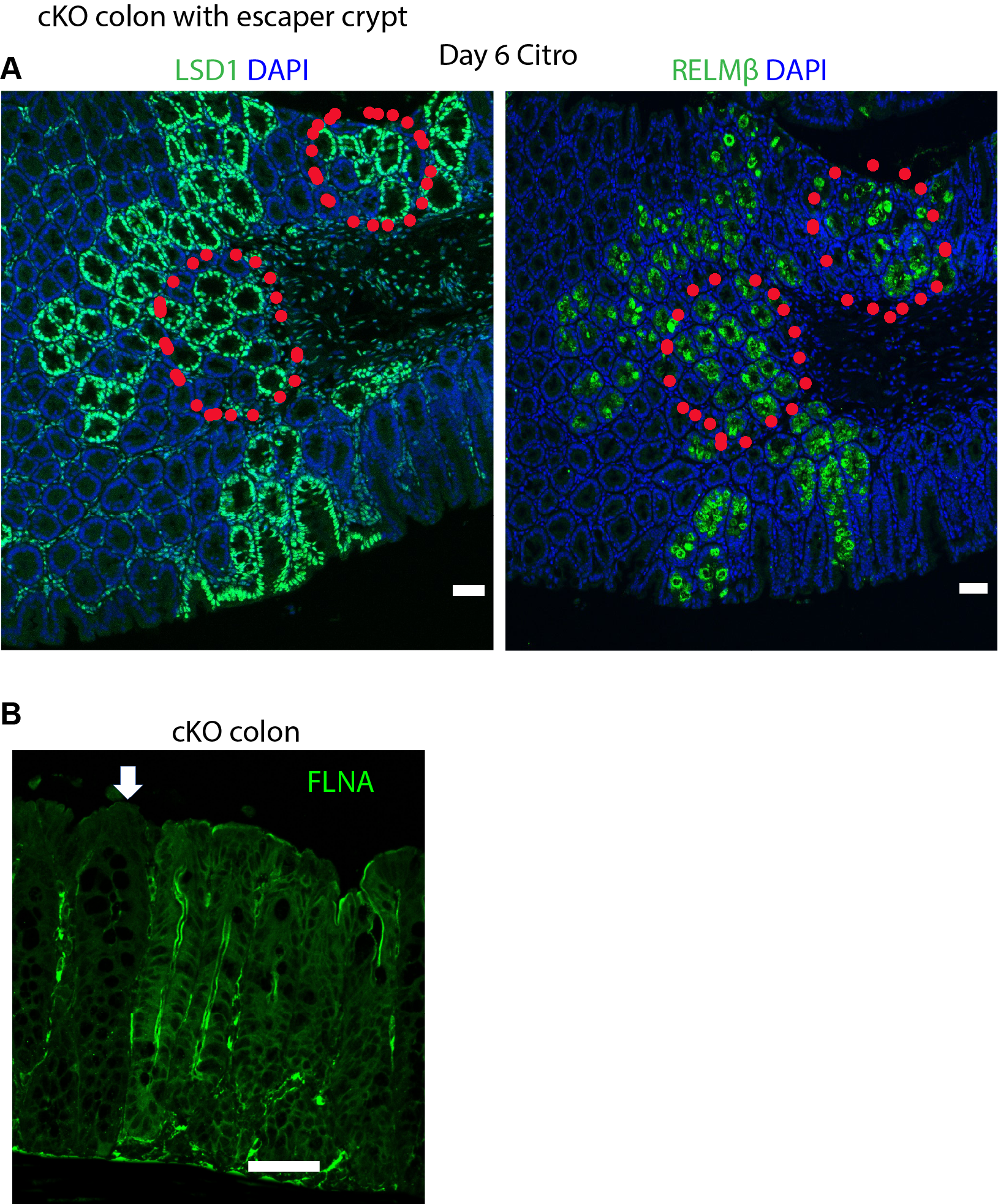

Supplement: S3 Fig — (A) Immunofluorescence staining to check co-localization of LSD1 (green) and RELMβ (green) in colon sections of cKO mice infected with C.rodentium for 6 days. DAPI (blue) was used as a counterstain. Original magnification: 10 X and scale bar- 50μm. (B) Immunofluorescence staining of FLNA (green) in colon sections from naïve cKO mice. White arrow indicates that there is no FLNA expression in WT escaper crypt. Original magnification: 20 X and scale bar- 50μm. (TIF) [file ppat.1009476.s003.tif]

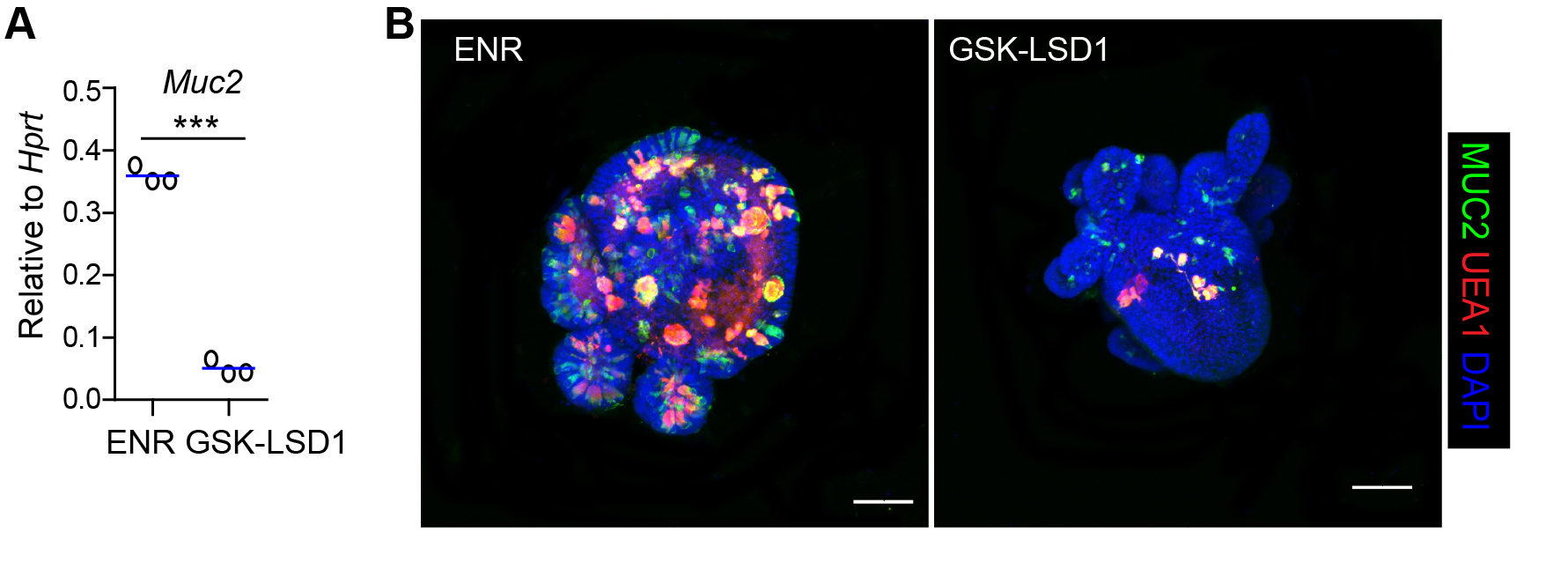

Supplement: S4 Fig — (A) RT- qPCR for Muc2 expression in organoids left untreated or treated with GSK-LSD1 for 4 days. Expression was normalized to housekeeping gene Hprt. (B) Immunofluorescent images of MUC2 (green), UEA1 (red) and DAPI (Blue) in WT organoids that were cultured in normal culture medium (ENR = EGF, NOGGIN, R-SPONDIN) or in ENR medium supplemented with GSK-LSD1 for 4 days. Scale bar is 50 μm. (TIF) [file ppat.1009476.s004.tif]

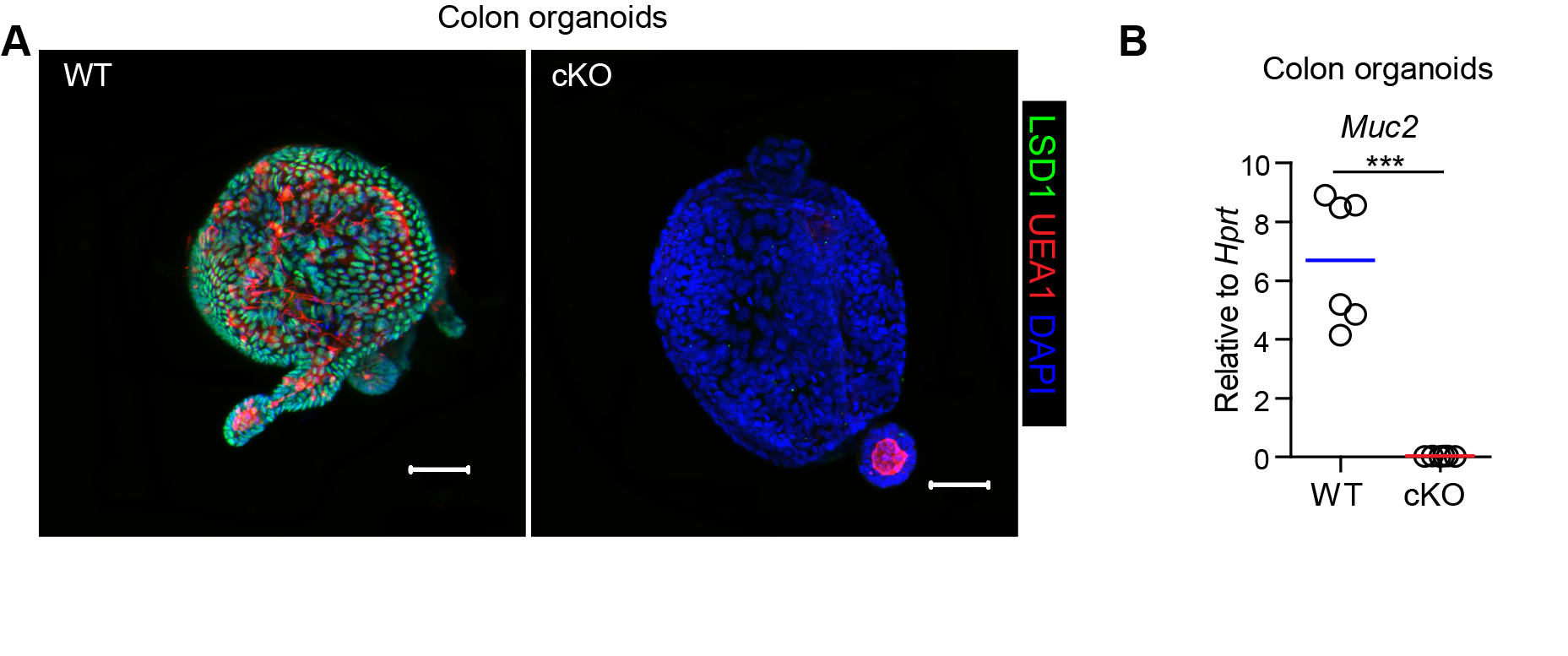

Supplement: S5 Fig — (A) Immunofluorescent staining of LSD1 (green), UEA1 (red) and DAPI (blue) in WT and cKO colon organoids was performed. Scale bar is 50 μm. (B) RT- qPCR for Muc2 expression in WT and cKO colon organoids cultured in ENR for 4 days. Expression was normalized to housekeeping gene Hprt. (TIF) [file ppat.1009476.s005.tif]
