# Supplementary material for: Intestinal-epithelial LSD1 controls goblet cell maturation and effector responses required for gut immunity to bacterial and helminth infection
Source: PLoS Pathog. 2021 Mar 31;17(3):e1009476. doi: 10.1371/journal.ppat.1009476 (PMC8041206; doi:10.1371/journal.ppat.1009476)
Supplement: S1 Table — (DOCX) [file ppat.1009476.s006.docx]

**Table 1.**

**Primers used for mRNA expression in a Real time qPCR assay.**

| **Gene name** | **Forward primer** | **Reverse primer** | **Detection method** |
| --- | --- | --- | --- |
| *Muc2* | cacgagacccaggaagtacag | gcaaagccactaactgcttgt | Taqman |
| *Retnlb* | cgtctcccttctcccactga | gacaaccatcccagcaggac | SYBR |
| *Il-22* | atgagtttttcccttatggggac | gctggaagttggacacctcaa | SYBR |
| *Il-17a* | agcagcgatcatccctcaaag | tcacagagggatatctatcagggtc | SYBR |
| *Ifng* | ggatgcattcatgagtattgcc | ccttttccgcttcctgagg | SYBR |
| *Reg3b* | ctcccaggcttatggctcct | tcatggagcccaatccaagt | SYBR |
| *Reg3γ* | ccgtgcctatggctcctattg | gcacagacacaagatgtcctg | SYBR |
| *Hprt* | cctcctcagaccgcttttt | aacctggttcatcatcgctaa | Taqman/SYBR |
