# Supplementary material for: Intestinal-epithelial LSD1 controls goblet cell maturation and effector responses required for gut immunity to bacterial and helminth infection
Source: PLoS Pathog. 2021 Mar 31;17(3):e1009476. doi: 10.1371/journal.ppat.1009476 (PMC8041206; doi:10.1371/journal.ppat.1009476)
Supplement: Attachment — Submitted filename: ParmarRebuttal.docx [file ppat.1009476.s008.docx]

**Response to reviewers.**

--------------------

<b>Part II – Major Issues: Key Experiments Required for Acceptance</b></br>Please use this section to detail the key new experiments or modifications of existing experiments that should be <u>absolutely</u> required to validate study conclusions.<br/><br/>Generally, there should be no more than 3 such required experiments or major modifications for a "Major Revision" recommendation. If more than 3 experiments are necessary to validate the study conclusions, then you are encouraged to recommend "Reject".<br/><br/>

Reviewer #1:

A major complicating factor/weakness is that as shown S Fig 1 the KO mice actually express significant levels of LSD1 in the colon. Thus it makes it quite difficult to dissect the mechanisms underlying the phenotypes described. The authors do not really address this point in any way.

*We agree with the reviewer this is an important point, and intrinsic to this mouse model that is very commonly used (and this feature is not often reported). We now present a more thorough examination of this in Fig. S1 and discuss it in lines 99- 101. In addition, we use it in our advantage in the Citro experiment; we observe Relmb+ patches, supporting our claim it is an epithelial defect due to Lsd1 deletion, not an immune response defect, new in Fig S3A). Similarly are the changes in FLNA we see in WT patches (new Fig. S3B)*

I am not sure that the colitis model is required here and part of a pathogen focused study. It shows a phenotype different to previous work looking at irradiation, but so do the two infection models used here. They should be sufficient.

*We agree with the reviewer, and as part of the refocus of this story we have taken out the DSS data, instead we have expanded on the analysis of the infection models.*

The two infection models used generate different types of immune response in the large intestine and thus different parameters are assessed, although it would have been useful to have a comparison of some of the parameters consistent across both models. e.g. goblet cell counts, proliferative counts, crypt lengths etc.

*We appreciate this comment by the reviewer and think it’s a good suggestion. We therefore have now included new data on crypt length, and Relm-beta IF and quantification for both infection models (new Fig. 3F&G and 4E&F).*

It is clear from the figures that the pathology changes quite differently in the KO mice - some more extensive analysis would help clarify things. In the worm model, is the worm loss just a delay or do the worms never the gut. Only one time point is examined.

*Unfortunately, our lab does not have the Trichuris model in place (this was done at Oudhoff’s previous institution), so we cannot address the question if it is a delay or not. However, as part of the control that was omitted in the initial submitted manuscript, we included Rag1KO mice (that never clear), and we now include that data. We observe similar worm counts in LSD1 cKO mice and Rag1KO mice, suggesting that it will lead to a chronic infection, which is in line with the extensive induction of IFN-gamma in cKO mice as well as the elongated crypts that is also characteristic of a chronic infection.*

The epithelial/basement membrane detachment assay is carried out on small intestinal tissue. How is this relevant to the rest of the paper which is concerned with the colon?

*We have now excluded this data from our manuscript as part of the refocussing of the story towards goblet cell effector responses. We find (at day 6 p.i.) there are no differences in FITC-dextran leakage in C. rodentium infected mice (new data requested by Reviewer 4). Thus, we do not think the loss of epithelium (which we observed at later stages in both the DSS and Citro models) is causing the early pathology we are observing, which is now the focus of the story. We still think the detachment of the epithelium is important, and is affected later in the DSS and Citro disease models – but due to the timing of the initial pathology it dilutes our story somewhat, therefore, we focus on goblet cell responses.*

There is considerable variation in the responses in many of the figures presented. Although the statistical tests have been carried out the data is far from convincing in some instances. In some cases there seem to be data from two experiments but in others this does not seem to be the case. There is no mention of the sex of the animals used. Was the same sex used throughout all experiments? If not, then at the very least this needs to be mentioned.

*We apologize for this, we have now increased the n by repeating some of the experiments (also requested by Reviewer 3), which makes the data more robust. We also note the sex of the animals in the methods. The data presented is pooled from several experiments using available littermates (both males and females).*

Reviewer #2: - The experimental evidence herein provided by the authors suggest a model in which LSD1 putatively controls enhancers of genes that shape the cytoskeletal organization of intestinal epithelial cells, which is needed for both cell attachment and cytokine-driven goblet cell effector responses after infection. This must be adressed by performing whole-genome bisulfite sequencing (WGBS) on colonic crypt Intestinal Epithelial Cells from wild-type and mutant mice. Alternatively, this can be adressed by a time course of chromatin immunoprecipitation followed by next-generation sequencing (ChIP-seq) profiling on purified intestinal epithelial cells.

*We agree with the reviewer that an additional technique showing that LSD1 controls putative enhancers of ‘cytoskeletal’ modulators would be useful. However, WGBS would measure DNA methylation, therefore, we opted to perform ATAC-sequencing to see if Histone methylation changes putatively done by LSD1 (i.e. different in KO compared to WT as was previously done by H3K4me1 ChIP) also results in differences in openness of the chromatin (by ATAC-seq, newly performed now in this study). We propose that this is equally or perhaps more informative than the proposed WGBS. We have performed ATAC seq comparing WT and KO crypt cells, and indeed find that overall open chromatin (by ATAC) associates with sites that have significantly increased H3K4me1, including at sites controlling cytoskeletal modulators such as shown for the Flna locus. This new data is presented in Fig. 6D-6E, and described in lines 241- 248.*

To test if altering the cytoskeleton would also allow for IL-22 mediated goblet cell effector responses, the authors treated cKO organoids with a combination of IL-22 and Y-27632, an inhibitor of ROCK signalling that is commonly used to manipulate the cytoskeletal network. Similar experiments must be performed on organoids derived from the colon of wild-type mice that were treated or not with a LSD1 inhibitor.

*We performed a set of new experiments trying to identify how LSD1 allows for appropriate goblet cell responses, including the experiments suggested here. We now also show that IL13+IL22 induces Relmb (whereas they don’t separately) in new Fig. 5K. In addition, we observe that Y-27632 treatment also in part allows for goblet cell effector responses in long term GSK-LSD1 treated organoids (new Fig. 7I&J).*

Reviewer #3: 1/Many of the experiments have not been repeated and graph are presented with only 3 or 4 replicates. Despite the fact that the differences are quite clear, it would be nice to be sure the phenotypes repeats and for some of the figures the dispersion of the datapoints with so few of them is insufficient to draw conclusions (particularly for Fig 1F, Fig 3EFig 4C, Fig 5C, Fig 6B).

*We have repeated experiments with new animals / organoids to increase the n to tighten the data, as was also suggested by reviewer 1. We now present all the data points available pooled from multiple experiments, including Figs. 1F, and new 2E, 4C and 5B, which correspond to the reviewer’s noted figure panels.*

2/Figure 6 :  Number of goblet cells are lower in cKO, it is difficult to conclude whether the absence of MUC2 induction in the KO after  IL-22 and DAPT is due to a change of activation or to the lower number of goblet cells. Presenting the data as MUC2+cells/organoids or better per area or organoids ( indeed it seems that the cKO organoids might be smaller than their WT counterparts on the images) might help clarify this issue. Another solution would be to present the data as MUC2/ UEA1 ( MUC2 production per goblet cells)

*UEA1, in our hands, is not always a reliable marker for goblet cells, especially in organoids (i.e. we have MUC2+ cells that are UEA1-). However, MUC2 is often secreted, so we cannot easily define/count MUC2+ cells. Therefore, we agree with the reviewer that MUC2 levels / organoid area is best, which is what we now present as data points throughout the manuscript. We now additionally present data on organoid size, including the responses to cytokines, DAPT, and the Rock inhibitor. New Figs. 5I and 5J.*

3/Figure 7 : Lines 333-346 : Following the hypothesis of the authors that the cytoskeleton is important for MUC2 driven IL-13 response in cKO and that altering the cytoskeleton could circumvent this issue  after IL-22 stimulation, then one would have expected the data shown in J to be MUC2 and not RELMb, considering that RELMb is not induced similarly in WT and KO contrary to MUC2. Could the authors explain their choice ? What results are obtained for MUC2 after Y-27632 treatment?

*These are excellent points raised by the reviewer: Because of our issues with MUC2 staining (see also above) – we decided to do RElMb as this was a much better quantifiable marker (number of + cells / organoid area). In addition, IL-22 is a much stronger inducer of Relmb than inducer of Muc2. Together, this was our reasoning.*

*Nevertheless, we have performed new experiments showing that, irrespective of IL22, iROCK slightly induces goblet cells (MUC2) in new Fig 7C and 7D, which further supports our hypothesis that LSD1 restricts goblet cells in part due to ‘cytoskeletal restrictions’. This is thus in contrast by the lack of MUC2 induction by DAPT in KO organoids (normally a much more potent inducer of goblet cells).*

Reviewer #4: 1.	More data is needed to reinforce the authors’ findings on decreased cell attachment and tissue delamination. I suggest high resolution confocal images of tissue slices stained for junctional/adhesion markers (e.g. E-cadherin and ITGA5) and actin, to better represent morphological changes in the crypt epithelium, and/or epithelial delamination. Also, upregulation of filamin A and ITGA5 needs to be better documented by immunofluorescence staining of the tissue (only filamin A staining in organoids was provided).

*In the experiments performed due to point 2 from this reviewer, we find no difference in FITC-Dextran uptake – therefore we don’t think the pathology (especially at this early time point) upon the citro infection is due to a breach of barrier. Although we think the delamination of the tissue is important later, we prefer to focus the story on goblet cell effector responses. Nevertheless, we have performed the Filamin-A staining (in vivo and in vitro in response to GSK-LSD1 treatment, data in new Fig 6H, 6I, S3B) as we propose that the cytoskeleton affects goblet cell differentiation and responses. We also did staining for E-cadherin in WT and cKO colon sections and did imaging at higher magnification (63 Objective, see below) We observe some differences, but it is difficult to assess if this is due to a lack of goblet cells, or a direct effect of loss of LSD1. Unfortunately, the Itga5 ab we ordered did not show any specific staining compared to the background. We therefore also took out the Itga5 RNA/ChIP data.*

*Fig.* Immunofluorescence staining of E cadherin (green) and DAPI (blue) in colon sections from naive WT and cKO mice. Original magnification is 63 X and scale bar is 10 µm.

2.	In addition to cell detachment assays which were performed (but not quantitated), intestinal permeability assays in vivo (FITC-dextran assay) are required in order to assess the level of the intestinal barrier breach, i.e. its functionality, which is a highly relevant experiment considering the pathophysiological scope of this study.

*We thank the reviewer for this important suggestion, we have performed this and do not find differences (New Fig. 2G). Thus, the pathology at the early timepoint (d6 p.i.), is likely due to the absence of Goblet cell effector program, which is now the main focus of this study.*

3.	A more thorough discussion is needed to help understand how LSD1-KO-induced upregulation of filamin A and ITGA5 (or other adhesion and cytoskeleton-related genes), could lead to observed delamination, colitis and inflammation. Similarly, the statement that LSD1 controls cytoskeletal-mediated cell identity (from the title) is rather bold, and definitely requires more experiments or some toning down. I believe authors are referring to experiments in Fig. 7I, with the ROCK inhibitor. Cell identity was not really confirmed by this experiment, and the effect of ROCK inhibition/cytoskeletal disruption was vaguely explained. I am not certain whether these results, as they are currently presented and discussed, benefit the paper’s central conclusions.

*We agree with the reviewer and have reshaped the title and our story towards goblet cell differentiation and effector responses, without claims regarding cell identity.* *Furthermore, we have extended the studies on ‘cytoskeletal’ control of goblet cells (Figs. 5-7) to focus our work and have rewritten these sections for clarity.*

--------------------

<b>Part III – Minor Issues: Editorial and Data Presentation Modifications</b></br>Please use this section for editorial suggestions as well as relatively minor modifications of existing data that would enhance clarity.<br/><br/>

Reviewer #1: (No Response)

Reviewer #2: Not applicable

Reviewer #3: Figure 1: F, in the legend, “the line represents littermate controls”. Are the littermate control of the cKO, WT ? In the graph, the lines look like the mean of the groups.

*Apologies for the confusion, the lines are indeed the mean. We breed Cre- X Cre+, so the WT and cKO littermates are born from the same mother and kept in the same cage (males and females separate).*

Figure S2A: the scale bar is missing and the WT and KO don’t seem to be at the same scale. Please use some arrow to point to the differences between the tissue.

*We have removed the DSS experiments from the story in agreement with Rev.1*

Figure 3: The authors states that the immune response to Citrobacter is not atypical. However, the IL17a gene expression, seems to have a trend toward a decrease, with only one outlier pulling toward non significance. It would be really important to have more data points here to ensure whether the Citrobacter response seen in the KO is really independent from the immune response.

*We have repeated the Citro d6 experiment to make sure our conclusions are valid. New, combined data is presented, and we find that immune responses are very similar (no significant differences).*

Figure 5 : “ENR” is defined in figure 7, but used in figure 5

*Thank you for pointing this out – fixed.*

Lines 254-262 : This section should part of the discussion rather than the results

*We have moved this to the discussion.*

Figure 6 :

-	IL=13 induction of MUC2 is unaltered in KO, while DAPT do not cause MUC2 induction in the KO. Does this mean that IL-13 driven response is NOTCH independent ? Is this known?

*We think this is an important point raised by the reviewer. We show that DAPT doesn’t induce MUC2 induction in KO organoids, but IL13, that also relies on Notch for goblet cell differentiation, is able to. We propose that IL13 also induces a cytoskeletal change (well established in B cells and macrophages (e.g. McWhorter et al, PNAS, 2013), and this allows for notch mediated differentiation, we have clarified this in lines 259- 267.*

-	Figure 6Fand G : the images chosen in F are poorly representatives of the differences depicted in G.

*Now we have fixed this figure. We are showing RELMB and beta-catenin hoping this makes these images more clear.*

-	In the text, the authors state that there is no significant difference between MUC2 induction after IL-13 in WT and KO, this stat should be shown on figure 6G

*We have changed this figure and altered the text.*

-	Figure 6H and I : like for the previous point, the images depicted in H are poorly representative of the data depicted in I (in particular IL-13 seems to induce way more RELMb than IL-22 in the WT)

*We have fixed this figure as well, for RElmb we are quantifying number of cells / organoid area not fluorescent levels like with MUC2. Of note, in related unpublished work we also find by gene expression that IL22 is a stronger inducer of Retnlb expression (see graph below), which is very similar to our quantifications of WT organoids in Fig. 5H*

Fig : WT organoids were treated with IL-13 (10 ng/ml) and IL-22 (10 ng/ml) for 72 h and RNA seq was performed. TPM- (Transcripts Per Million) are shown for *Retnlb*.

-	G and H : despite the fact that the same type of quantification is done in those two figures, the results are presented differently (AU versus +cells/organoids). Please homogeneise the way the fluorescence data are shown and add a section in the Material and methods explaining the quantification.

*Apologies, we have made all these figures now consistent and described this in the material and methods; Muc2 we quantify signal / organoid area, but our preference, which we can do with Relmb, is #cells / organoid area.*

Figure 7:

-	7C : There is not enough informations in the Methods or the legends as to how the images in C are obtained. Is this cytospins ? Is the same volume plated between WT and cKO ? without the explanation, the change of density observed in the images cannot be interpreted.

*Based on the other reviewer comments, we have taken out this data, also to focus the story.*

-	7D : TPM is not defined

*Apologies, and thank you, changed.*

-	7F and J : once the again the fluorescence data are presented differently (AU versus +cells/organoids) without any justifications.

*Apologies, we have made all these figures now consistent and described this in the material and methods.*

Figure 7:

-	Is the Y-27632 treatment affecting the morphology of the organoids, on the representative images it seems that there is less crypts, and that the organoids are more “spheroid”. Could this contribute to the phenotype reported in J. Once again presenting the data as RELMb+ cells/goblets cells (UAE1) might help clarify whether it is a cell number or an activation issue.

*We have extended this story line and also show the quantification and qualification (% of spheroids) of these organoids (new Fig. 7F).*

Reviewer #4: •	Methods: the section on organoid isolation and culture is completely missing.

*Apologies, this is obviously one of the main methods that should have been in the original manuscript, included now. Please see materials and methods sections lines 426- 446.*

•	Methods: please specify how immunofluorescence experiments in organoids were quantified, especially normalization to cell number/organoid size (Figs. 1C, 6F-I, and 7I).

*Addressed, see also points to reviewer 3. Muc2 we quantify signal / organoid area, but our preference, which we can do with Relmb, is #cells / organoid area. Methods are described from line 479 onward*

•	line 93; also line 268-269: make a clear distinction with the previous study—which organoids were used, small intestinal or colonic? The authors should be more specific about the origin of organoids throughout the text, considering they are often citing their previous study on the small intestinal epithelium.

*Our organoid work here is done from small intestinal crypts. This alleviates the issue with reduced genetic recombination in the colon noted by Reviewer 1. However, and more importantly, small intestinal organoids can grow without WNT supplementation (colon organoids can’t). The WNT supplementation results in lack of differentiation and the presence of only progenitors in the organoids, which hampers studying goblet cells and cytokine-response assays such as described in our this study.*

•	line 303-304: “In our previous work we found that the transcriptomic profile of cKO small intestinal crypt cells is fetal-like (Zwiggelaar et al., 2020)”. It also appears in line 370: “Indeed, in a previous report …”. I am unsure if unpublished work, i.e. preprints, should be described as “previous work”.

*This important work on which our study builds is now published in Science Advances, and appropriately referenced.*

*https://advances.sciencemag.org/content/6/37/eabc0367.abstract*

•	line 135: statement “like it is upon damage induced by irradiation” misses a reference.

*As suggested by Rev. 1, we have taken out the DSS data.*

•	Fig. 7C – quantitation is missing.

*We have taken out this research story line; it likely has little effect on the initial pathology (new FITC-Dextran assay), and it is better to have 1 focus in this study*

•	Fig. 7B: why is there upregulation in untreated organoids? A more thorough discussion of results is needed.

*Apologies for not making this clearer, the comparison is within the separate groups (i.e. crypts (WT and cKO) and Organoids (untreated vs. GSK-LSD1). We have rephrased this part (lines 238-240)*

•	Fig. 7I: Related to Fig. 6G-I, it would be interesting to see what is happening with Muc2 – is it also being “rescued”?

*We have now performed new exps, indeed Muc2 is slightly induced in cKO organoids upon iROCK inhibition. And this is shown in figs 7C and 7D.*

•	Scale bars should be without label in the figures. You should indicate scale bar length in the figure legend.

*Thanks for the suggestion. Yes, we have change this throughout all the figures, and note the scale bar size in the legends.*

•	Abbreviations such as TPM (Fig. 7D) and IGV (Fig. 7G,H) should be explained in the legends or methods.

*We have now explained these abbreviations at first use.*
