# Supplementary material for: Intestinal-epithelial LSD1 controls goblet cell maturation and effector responses required for gut immunity to bacterial and helminth infection
Source: PLoS Pathog. 2021 Mar 31;17(3):e1009476. doi: 10.1371/journal.ppat.1009476 (PMC8041206; doi:10.1371/journal.ppat.1009476)
Supplement: Attachment — Submitted filename: Parmar Rebuttal second revision.docx [file ppat.1009476.s009.docx]

**Response to reviewers. Second revision**

Reviewer #3: 1. Assessing goblet cell differentiation based only on immunostaining for Muc2 and RELMB, PAS staining or lectin-binding capacity (UEA1), is problematic. As the authors themselves stated in their response to reviewer 3, (2/Figure 6) “MUC2 can be secreted, and thus these cells cannot be counted; UEA1, in our hands, is not always a reliable marker for goblet cells, especially in organoids (i.e. we have MUC2+ cells that are UEA1-)”. Also, the relationship between Muc2 and RELMB does not seem to be trivial (RELMB was dramatically upregulated in Muc2-/- mice; see Morambudi et al. 2016; PMID: 26813339). Moreover, judging from the image of the colon crypts (63X; E-cad, DAPI) that was provided in response to my concerns in the previous revision, many cells with goblet-like morphology are still present in the cKO. Thus, considering the complexity of assessing cell identity/differentiation, my suggestion is that the focus of the study should remain on goblet cell effector responses in LSD1-KO and infection models, which are well established in this study, and not in a role of LSD1 in goblet differentiation, for which the evidence is not enough.

*We thank the reviewer for their remarks. We agree that the strongest effect we observe is an absence of effector responses, perhaps the differences in naïve mice can best be described by an impairment in* ***goblet cell maturation****, by using this term we avoid the putative differentiation into goblet cell precursors while still noting the differences in MUC/UEA1 we are observing in naïve mice.*

*We therefore have changed ‘differentiation’ with ‘maturation’ throughout the manuscript, including in the title ‘maturation and effector responses’ instead of ‘differentiation and effector responses’.*

2. Authors should address the role of LSD1-KO in colon organoids, in order to be consistent with the organ and epithelial tissue they are addressing in their in vivo experiments. Authors’ reasoning to use small intestinal (SI) organoids as a model in this study was based on less efficient recombination of LSD1-KO in the colon and, more importantly, the fact that colon organoids culture requires Wnt, which results in incomplete differentiation. According to Sato et al. 2011a (PMID: 21889923), Wnt can be removed (“differentiation medium”) which then allows for complete differentiation. Please note that Sato et al. assessed epithelial cell differentiation in cultured colon organoids by measuring mRNA expression of various markers (Muc2, ChgA, Alpi..) and thus did not solely rely on immunostaining or lectin binding.

*We agree that main experiments in colon organoids are important to validate our work. We isolated colonic crypts and maintained colon organoids in Wnt +ENR (WENR) media, which supported both WT and cKO colon organoids (See Figure below). After passage, we switched to ENR for 4 days of differentiation and performed MUC2 immunofluorescent staining and Muc2 qPCR experiments. We found that MUC2 positive cells are significantly lower in cKO colon organoids, and a similar reduction is found by qPCR (****new Fig. 5C, 5D, S4C, S4D****). Described in line 197 – 199.*

Figure: WT and cKO colonoids were grown in 50 % WNR for 4 days. Bright field images were acquired using EVOS microscope. Magnification: 2 x objective.

3. Authors claim that “FILAMIN A, a key protein in mechanosensing and mechanotransduction (references are missing), is a direct target of LSD1 demethylase activity” yet they don’t try to functionally validate this proposed role of filamin A in the colon, so this seems to be an exaggeration. I suggest that the authors refrain from such statements unless they are able to tackle and explain the role of filamin A in this context, assuming there were more candidates coming from the screen. As it stands, filamin A and its increased expression in this study represents an example confirming their RNAseq screen and the follow-up analysis.

*We indeed think of FILAMIN A as an example as one of the ~65 genes associated with actin / cytoskeleton / junction / adhesion (Fig. 6B, 6C) that could affect mechano-aspects. We meant that Flna is likely directly controlled by LSD1 demethylase activity (affecting H3K4me1 and chromatin accessibility), not that it would be ‘the’ key mediator of goblet cell maturation within our LSD1 studies. Nevertheless, we agree that this was not clear enough. We therefore changed lines to tune down/avoid claims with regard to this:*

*Abstract now stating that ‘We propose this is may be …’, rather than ‘In part,…’*

*Line 262 ‘partially control’ instead of ‘define’*

*Line 286 ‘may affect’ instead of ‘affect’*

*Line 310 ‘Further studies….’*

4. Authors used ROCK1 inhibitor to perturb “cytoskeletal organization”, which in itself sounds very vague. They should explain the rationale for this approach and discuss the results of this treatment in the context of what is known or proposed for this kinase inhibitor, including the maintenance and differentiation of stem cells, in addition to the well-studied effect on actomyosin contractility, which was not mentioned or discussed either.

*We apologize this was not clear previously, we base this work on Petersen et al (PMID 29935151). They used the ROCK inhibitor in vivo and in organoids and found increased numbers of secretory cells (including goblet cells). We have now included this more prominently and clearly in the text. Line- 274 – 277.*

**Part III – Minor Issues: Editorial and Data Presentation Modifications**

Reviewer #3: 1. Line 452: Methods: “Bright field imaging and quantification of intestinal organoids” - instead of referring to their preprint, authors should explain in this paragraph how their images were quantified.

*Yes, we agree, we now describe the steps for quantification of intestinal organoids using the script in this manuscript.*

2. Line 100-101: LSD1 expression in colon cKO is not quantitated.

*Thanks for the suggestion. We have included quantified LSD1 expression percentage in WT and cKO mice in the supplementary fig S1B.*

3. Fig. 1G, H, S2B: Western blot should be repeated and quantified (gel band densitometry). Authors need to clearly assess whether STAT phosphorylation response is the same or weaker upon IL-22 induction in cKO. The confocal images of organoids in S2B were not quantified either.

*We have repeated the Western blots and incorporated band intensity quantifications in the main figure 1H. Below are the new repeated western* blot results showing similar STAT3 phosphorylation. We have decided that we remove the immunofluorescence images of pSTAT3 and STAT3 from supplementary figures S2B as they don’t provide new insights.

Figure: Western blot for pSTAT3, STAT3 and GAPDH in organoids repeated from 2 different additional mice.

4. Line 131-132: increased mortality and weight loss are relatively mild, especially at day 6 pi. Why not looking at later stages?

*We observe mice begin losing weight at day 3 or 4, and mortality is between day 6 and 12. We picked day 6 as at this time point there are clear changes in weight (Fig. 2B), but we reasoned would be early enough to not pick up secondary effects, as we wanted to identify the cause for disease in these mice.*

5. Line 137-139: the effects of Citrobacter infection on the gut barrier permeability should be more clearly explained in the text. The barrier permeability was increased upon Citro infection, but there was no significant difference between the ctrl and the KO at day 6 pi.

*We have now clarified this, indeed Citro infection induces permeability, but not different between WT and cKO mice. line…138- 140.*

6. Figs. 2-3C, F, G: Authors should provide data also for naïve crypts (ideally for both wt and cKO).

*We try to have naïve controls in our infection exps., especially for the analysis that easily allows higher throughput (ELISA, qPCR), and for this we often use just WT naïve mice (not both), for the labor intensive swiss roles / IF staining and confocal imaging we don’t commonly do this. We propose it’s best to keep the infection imaging just WT vs cKO infected in Fig. 3 and 4 for consistency, and have the naïve comparisons separate in Fig. 1, 5, and 6.*

7. Fig. 3D, F: Authors should be explicit on whether they analysed crypts from the same region of the colon, as crypt length varies significantly between different regions of colon (PMID: 24205248).

*Yes, the C. rodentium is distal colon, the T. muris is cecum, we included this information in the legend.*

8. Fig. S3A: These images are seemingly not from the same focal plane (or perhaps not the same region), so it is impossible to determine “colocalization”; also, “escaper” crypts should be somehow highlighted.

*We apologize for the confusion. For these imagines, the LSD1 and RELMβ staining’s were carried out on different glass slides, and that’s why the tissue localization is not 100 % same in both the sections (both are Rabbit antibodies and so this can only be done in sequential sections, thus, not exactly on the same plane). To make it more clear, we now highlighted the escaper” crypts with red color to show the similarly recognizable areas of LSD1 and RELMβ in cKO colon sections. And new revised figure has been put in the supplementary data.*

9. Line 182-183: can authors explain why the crypt length is different for WT and cKO between Citro and T. muris conditions (Fig. 3C vs 4G)?

*As the reviewer noted above, crypt length is very different depending on location in the colon. In addition, these two different pathogens have their own ‘favourite’ location (T. muris infects crypts in the caecum, whereas Citro ends up in the distal colon). Therefore, the sections from T. muris (Fig. 4G) are from the caecum (where the parasite infects), and Citro (Fig. 3C) is from distal colon. We have now noted this specifically in the legends.*

10. Line 197: “we found that goblet cell differentiation and maturation is much more
impaired in the colons of naïve cKO mice, as shown by reduced numbers (differentiation)…”. Authors should be more careful – they did not show numbers of goblet cells (they show number of Muc2+ and UEA1+ cells per crypt, as stated in Fig. 5B and C).

*In line with the first comment of the reviewer, we avoid the use of differentiation and use ‘maturation’ in naive conditions, and ‘goblet cell effector’ in infection experiments.*

11. Line 224: Authors used data from RNAseq performed on SI crypts cKO/WT and GSK-LSD1/untreated organoids and gene enrichment analysis, instead of performing this analysis on colon tissue/organoids. It should be explained why they did not analyse colon tissue/organoids.

*We provide the rationale that we anticipated that LSD1-controlled mechanisms would be similar between colon and small intestinal epithelium. Line 227-230. In addition, such RNA-seq experiments and analysis are costly and we did not have budgeted this in the funded studies.*

12. Line 259: hyphen missing (LSD1 controlled)

*Thank you, corrected.*

13. Line 263: "induce MUC2 expression" would be more correct.

*Thank you, corrected.*

14. Line 295: remove colitis, as those experiments were removed.

*Apologies, we corrected this*.

15. Line 316: GFI1-KO mice were shown to have decreased goblet cell numbers/maturation, and not a complete lack of – this should be corrected.

*Thank you, corrected.*
